# Experiences of mental health professionals supporting front-line health and social care workers during COVID-19: qualitative study

**DOI:** 10.1192/bjo.2021.29

**Published:** 2021-03-23

**Authors:** Jo Billings, Camilla Biggs, Brian Chi Fung Ching, Vasiliki Gkofa, David Singleton, Michael Bloomfield, Talya Greene

**Affiliations:** Division of Psychiatry, University College London, UK; Division of Psychiatry, University College London, UK; Division of Psychiatry, University College London, UK; Division of Psychiatry, University College London, UK; Division of Psychiatry, University College London, UK; Division of Psychiatry, University College London, UK; Traumatic Stress Clinic, Camden & Islington NHS Foundation Trust, UK; National Institute for Health Research University College London Hospitals Biomedical Research Centre, UK; and University College London Hospitals NHS Foundation Trust, UK; Division of Psychiatry, University College London, UK; and Department of Community Mental Health, University of Haifa, Israel

**Keywords:** COVID-19, mental health professionals, qualitative research, front-line workers, psychosocial interventions

## Abstract

**Background:**

The coronavirus disease 2019 (COVID-19) pandemic is having a well-documented impact on the mental health of front-line health and social care workers (HSCWs). However, little attention has been paid to the experiences of, and impact on, the mental health professionals who were rapidly tasked with supporting them.

**Aims:**

We set out to redress this gap by qualitatively exploring UK mental health professionals’ experiences, views and needs while working to support the well-being of front-line HSCWs during the COVID-19 pandemic.

**Method:**

Mental health professionals working in roles supporting front-line HSCWs were recruited purposively and interviewed remotely. Transcripts of the interviews were analysed by the research team following the principles of reflexive thematic analysis.

**Results:**

We completed interviews with 28 mental health professionals from varied professional backgrounds, career stages and settings across the UK. Mental health professionals were motivated and driven to develop new clinical pathways to support HSCWs they perceived as colleagues and many experienced professional growth. However, this also came at some costs, as they took on additional responsibilities and increased workloads, were anxious and uncertain about how best to support this workforce and tended to neglect their own health and well-being. Many were professionally isolated and were affected vicariously by the traumas and moral injuries that healthcare workers talked about in sessions.

**Conclusions:**

This research highlights the urgent need to consider the mental well-being, training and support of mental health professionals who are supporting front-line workers.

The coronavirus disease 2019 (COVID-19) pandemic is having a well-documented impact on the mental health of front-line Health and Social Care Workers (HSCWs).^1,2^ In anticipation, a national ‘call for action’ was made to the UK mental health community to urgently provide additional support for front-line workers’ well-being.^3^ The experiences, views and needs of the mental health professionals called upon to provide this support have, however, so far been overlooked, both in the UK and globally. In this study we set out to redress this gap by qualitatively exploring mental health professionals’ experiences of supporting front-line HSCWs during the pandemic and how providing this support has impacted on them.

## Method

### Participants and procedures

All procedures were approved by the University College London Research Ethics Committee (Ref. 18341/001). We recruited mental health professionals purposively through Twitter and by snowball sampling via mental health colleagues. We deliberately sought to recruit a wide range of participants, including different mental health professions, career stages and geographical regions, to access a diverse range of experiences and views.

Potential participants were invited to contact the first author via email and sent the study participant information sheet and consent form by return email. Interviews were completed by three graduate master's students and a psychological well-being practitioner (PWP), all of whom received training and supervision from the first author (J.B.). Written informed consent was obtained from all participants prior to taking part in the interviews.

Interviews were conducted by telephone or online video call and guided by a semi-structured interview guide (see supplementary Appendix 1 available at https://doi.org/10.1192/bjo.2021.29). The interview guide was drafted collaboratively by the research team, in consultation with our expert reference group, comprising experts in psychological trauma and National Health Service (NHS) well-being leads, and including professionals with lived experience of mental health difficulties.

Interviews were audio recorded and transcribed verbatim by the interviewer who conducted the interview. Potentially identifying information about the participants such as their place of work were removed from the interview transcripts to protect participants’ anonymity. Pseudonymns are used throughout.

The authors assert that all procedures contributing to this work comply with the ethical standards of the relevant national and institutional committees on human experimentation and with the Helsinki Declaration of 1975, as revised in 2008.

### Analysis

We followed the principles of reflexive thematic analysis^4,5^ throughout this study. We sought immersion in the data by reading and re-reading all the transcripts, reflecting on the interviews and discussing emerging themes in research team meetings. J.B. and the four interviewers each independently coded two transcripts to derive an initial list of potential codes. This coding frame was reviewed and agreed through discussion in the team. All transcripts were then imported into NVivo Pro V12 and coded into the provisional coding frame, which was further extended and revised with the coding of subsequent transcripts. All coding was inductive, derived from the data, and not pre-determined by pre-existing theories. A final set of themes was then developed from the coded data and refined with feedback from participants, mental health colleagues and our expert reference group.

### Ethical issues

We were aware of potential research burden on mental health professionals who were already taking on new roles and responsibilities in their work with front-line HSCWs. We sought to minimise this by casting our recruitment net widely so as not to overly impose on particular services or localities and ensured that all participation was entirely voluntary. Given the emotive nature of this work, we included information about support services that mental health professionals could also access in our participant information sheet and during the interview. We also sought to protect our research team from the potential impact of hearing about mental health professionals’ distress through training and regular supervision.

### Quality

We have adhered to the highest standards for conducting and writing up qualitative research throughout this study, drawing on existing frameworks for quality in qualitative research: including the Standards for Reporting Qualitative Research Framework^6^ and specific guidance for quality practice in reflexive thematic analysis.^7^

In qualitative research, we place less emphasis on reliability and generalisability but attend more to validity, transferability and trustworthiness.^8^

To increase the validity of our results, we included multiple researchers in the processes of data collection, coding and analysis, to challenge our own assumptions and identify potential ‘blind spots’ that any one of us might have regarding this subject. We presented our preliminary findings to mental health colleagues in a variety of forums to discuss the face validity of emerging themes. Two mental health professionals who took part in the interviews provided feedback on our analyses as a form of member checking.

We have not sought to achieve a representative sample of participants with the intention of generalising our results to all mental health professionals in the UK. Rather, in keeping with the epistemology of qualitative research, we have deliberately tried to include as varied a group of participants as possible in order to explore the potential diversity of experiences and views, and increase the potential transferability of our findings.

To increase the trustworthiness of our interpretations, we have sought to be transparent about the research team conducting the study and the lenses through which we have viewed this data. We provide quotes from participants to illustrate and evidence our analyses.

### Reflexivity

The research team behind this study is made up of a diverse group of researchers, including different career stages and clinical specialities. We also represent a range of genders (three of the research team are female, and three male) and cultural groups (including White British, White European and Asian). The first author, J.B., is a consultant clinical psychologist and associate clinical professor with over 20 years of experience of working in the NHS with specialist expertise in trauma, mental health and well-being in high-risk occupational groups. C.B., B.C.F.C. and V.G. were all MSc students in clinical mental health sciences when this research was conducted. D.S. is a PWP and previous graduate of the MSc in clinical mental health sciences course. All volunteered to work as research assistants on this study. M.B. is a consultant psychiatrist and principal clinical research fellow with 19 years’ experience of working in the NHS. T.G. is a senior lecturer specialising in research on psychological responses to mass traumatic events.

As clinicians as well as researchers, J.B., D.S. and M.B. were also working with front-line workers during the pandemic. None of us were redeployed, but like many of the mental health professionals in this study, our work was redirected to the pandemic and its impact on the mental health of certain groups. In response to the pandemic, J.B., M.B. and T.G. co-founded and co-direct the COVID Trauma Response Working Group, a national group of expert clinicians and well-being leads, which was convened to provide trauma-informed and evidence-based guidance to support the mental health and well-being of high-risk groups. This multiplicity of perspectives confers some advantages in terms of our insight and deep knowledge on this subject. However, there are also potential disadvantages in our closeness to the topic. We have sought to address this throughout all stages of data collection and analysis by maintaining curiosity about our data, working closely as a research team and welcoming diverse and alternative views.

## Results

Twenty-eight mental health professionals volunteered and took part in the study. The gender, roles, settings and geographical locations of participants are shown in Table 1.

**Table 1:** Participant characteristics (*n* = 28)

Characteristic	*n* (%)
Gender	
Women	23 (82)
Men	5 (18)
Role	
Clinical psychologist	13 (46)
Trainee clinical psychologist	3 (11)
Psychological well-being practitioner	3 (11)
High-intensity cognitive–behavioural therapy therapist	2 (7)
Psychiatrist	2 (7)
Mental health occupational therapist	2 (7)
Counselling psychologist	1 (4)
Mental health practitioner	1 (4)
General practitioner (special interest in mental health)	1 (4)
Setting^a^	
General/acute hospital	12
Older adult hospital setting	2
Intensive care unit	3
Nightingale hospital^b^	3
Phone/online staff support	2
Care home	1
Increasing Access to Psychological Therapies service (primary care psychology)	7
Ambulance service	1
General practice	1
Geographical location by UK nation and region	
England	
London	15 (54)
South East England	1 (4)
South Central England	2 (7)
Midlands/Central England	3 (11)
North East England	1 (4)
Wales	1 (4)
Scotland	5 (18)

a. Several participants worked across more than one setting in response to the pandemic.

b. Specialist hospitals set up for coronavirus disease 2019 (COVID-19).

Most participants had been redeployed into new services or involved in setting up new treatment pathways for staff support. All experienced changes in their work practice; even participants who had a dedicated specialist role in supporting physical healthcare wards including intensive care units (ICU) experienced a change of focus from supporting patients to supporting staff. Most participants were working remotely, although some still had face-to-face contact with staff in hospital settings.

Interviews took place between 8 June and 23 July 2020, which corresponded with the early post-peak phase of the pandemic in the UK. Interviews lasted between 28 min and 1 h 55 min, although most interviews took between 40 and 60 min.

Analysis of the interview data identified six inductive themes, which are discussed in detail below. Pseudonyms are used throughout.

### Themes and subthemes

1. Stepping up:
  - motivation and purpose
  - learning and growth
  - additional responsibilities and increased workload.
2. Uncertainty, inconsistency and lack of knowledge:
  - anxiety and uncertainty
  - inconsistency of service provision
  - lack of specialist knowledge.
3. Blurred boundaries – colleagues or clients?
  - shared experience
  - confidentiality.
4. Isolation.
5. Self-sacrifice and subjugation of own needs.
6. Vicarious traumatisation and vicarious moral injury:
  - traumatic exposure
  - ethical and moral dilemmas.

### Stepping up

#### Motivation and purpose

The mental health professionals we interviewed were all, without exception, very motivated and driven to support HSCWs. They perceived themselves as part of the front-line effort and were keen to ‘step up’ and develop new services to help workers they perceived as colleagues. Doing so gave them a strong sense of purpose and they valued being able to do something meaningful to contribute.

‘*It has given us a sense of purpose, of something that we could do positively with the skills that we have during a time where everything looks really dark. I think it has been something to focus on during a real period of uncertainty which has been really great, it has felt good and important and purposeful to do.’* (Olly, trainee clinical psychologist, hospital)

Mental health professionals also appreciated feeling valued by their colleagues and the trusts in which they worked. Several commented on the raised profile and increased appreciation of psychological services.

‘*We as psychologists have been embraced by the department in a way that I have not experienced in the past. It felt that they needed us and that feels really quite special.’* (Lucy, clinical psychologist, hospital)

#### Learning and growth

For the most part, mental health professionals welcomed the challenge of setting up new clinical pathways and with that came opportunities for growth, development and learning. Participants talked about being able to apply transferable knowledge and skills, broaden their connections with colleagues and raise awareness of mental health and well-being. Many had taken on development and leadership roles.

‘*I think there's been a lot of growth and opportunity to develop as well. I've developed a lot of skills from this that I wouldn't have gotten from another placement or training roles.’* (Archie, trainee clinical psychologist, Improving Access to Psychological Therapies (IAPT))

#### Additional responsibilities and increased workload

With the rapid development of new services and treatment pathways came additional responsibilities and increased workloads. Although mental health professionals welcomed new challenges, embraced opportunities and were very motivated to help their colleagues, many also acknowledged that this way of working was not sustainable.

‘*One of the biggest challenges was trying to take on quite a substantial extra part of my role on top of what I already do…I was working an extra 2 h every time I was at work, which is completely understandable and needed to be done, but also not completely sustainable.’* (Faizah, clinical psychologist, hospital)

This appeared to be compounded by HSCWs working shifts, often requesting contact outside of normal working hours from mental health professionals. Emily, a clinical psychologist in ICU told us:

‘*I think the hard thing was the ICU staff obviously work night and day, not just 5 days a week and over weekends as well. So the really difficult thing was trying to maintain any kind of boundary over your own working life…there was always someone who wanted to speak to you who had been working all week and wanted to talk to you at ten o'clock on a Friday night.’*

### Uncertainty, inconsistency and lack of knowledge

#### Anxiety and uncertainty

Although mental health professionals felt a strong sense of motivation to support their HSCW colleagues, stepping into what was for many a new arena brought with it considerable anxiety and uncertainty. Many had never worked in physical health or social care settings previously, had little understanding of medical procedures and terminology, and few had direct experience of working with staff. Even those who worked alongside physical health wards and ICUs usually had more patient-facing roles and had not previously experienced such high rates of morbidity and mortality.

Several participants talked about there being neither established programmes nor protocols for supporting staff in this context, which led them to worrying about whether they were doing the right thing.

‘*Everything is uncertain…and I think am I doing the right thing? Is this what they need? There is no specific protocol. I felt deskilled.’* (Estelle, PWP, IAPT)

#### Inconsistency of service provision

Mental health professionals talked about drawing on a number of models and approaches to guide their work in this new area, including psychological first aid, active monitoring, psychoeducation, practical support, compassion-focused therapy, mindfulness, meditation and reflective groups. Many talked about phased-based and stepped-care models and most recognised the importance of not offering psychological interventions too early. However, there was not always clarity or agreement between different mental health professionals about what was the best course of action.

‘*It's been difficult to work with colleagues sometimes, people can have quite different visions of the ways in which they think we should deliver this support. It's tricky to navigate all the different opinions and people keep on doing their own thing, so it sounds like there's not much cohesion or unified response which is difficult.’* (Archie, trainee clinical psychologist, IAPT)

Although mental health professionals worked flexibly and creatively to adapt and develop new pathways, there was recognition that services set up rapidly were often not consistently coordinated or coherently promoted.

‘*I think it was all a bit hap-hazard, you know, by necessity, you know there wasn't an existing structure.’* (William, psychiatrist, hospital)

*‘Everybody's been getting on with their own, their own way of offering something, and I think sometimes that's felt a little bit disjointed…I've had lots of contact from lots of different people saying “we're trying to offer this” or “we're thinking of offering that” and I think it would have been amazing if that could have been pulled together more coherently.’* (Katrina, clinical psychologist, care homes)

#### Lack of specialist knowledge

Participants identified key areas of knowledge in which they felt they were lacking. Mainly this related to knowledge of health and social care settings and the needs of HSCWs specifically. Without formal training or previous experience in this context, many mental health professionals did not know what was normative in that culture or how to benchmark usual behaviour.

‘*Prior training on different populations like frontline staff and healthcare workers [is needed] because they come with very niche and specific difficulties like lack of self-compassion and that's not taught on the course at all. We're never taught to consider healthcare staff as needing support and being vulnerable to problems until it happens. I think for this role to go well, we need to think about having this more ingrained into training.’* (Archie, trainee clinical psychologist, IAPT)

Mental health professionals also acknowledged a lack of awareness between different support structures. They were often unsure of what other services were providing and several commented on a mutual lack of awareness between psychological therapy and occupational health services.

### Blurred boundaries – colleagues or clients?

#### Shared experiences

Mental health professionals talked about relating to HSCWs as colleagues, which seemed to lead to them feeling additionally compelled to care for them. This fuelled their motivation to do this work, but sometimes came at a cost to their own well-being, and boundaries could become blurred as their colleagues became their clients.

‘Y*ou really strongly identify with the people that you're supporting. Usually there is a degree of separation…but a lot of these people we are seeing are people we may have known beforehand, may have been colleagues or may have seen around in this small hospital. If you identify really strongly with someone it is really hard to resist a pull to fit one more appointment in between several others and to run yourself a bit ragged doing that…maybe you just want to give five percent more or do a little bit more…and I would link that to the fact that you know these people are very similar to yourself just in different roles.’* (Olly, trainee clinical psychologist, hospital)

Mental health professionals also identified with their health and social care colleagues directly through their shared experience of the pandemic, which further blurred boundaries.

‘*You can't really separate your job from the situation. Working through COVID and also being from a BAME [Black, Asian, minority ethnic] background and [talking about] how they've been impacted, you kind of think, oh how have I been impacted and how you relate to them. You have to work hard to be quite reflective and not overly identifying with what the staff are telling you in the wellbeing sessions.’* (Faizah, clinical psychologist, hospital)

#### Confidentiality

This blurring of boundaries was also manifested in issues with confidentiality. There were often not clear or confidential routes for HSCWs to access mental health support in the settings in which they worked alongside mental health professionals day to day. Mental health professionals gave examples of working alongside someone in a multidisciplinary team who they may be seeing for individual psychological support.

‘*It's quite a difficult position to be in because you know personal things about them, such as their mental health, how they're coping, their past mental health difficulties. When that's all over, how do you go about working with them when you know so much information about them and who they are? It really blurred the boundaries between my work and role as a psychologist and knowing so much more about your colleagues.’* (Faizah, clinical psychologist, Hospital)

This could raise further ethical and risk management dilemmas about material HSCWs disclosed in sessions. Shared experience therefore seemed to be both an enabler and a barrier to the support offered by mental health professionals.

### Isolation

Unlike their health and social care colleagues who were working in larger teams in hospital or care home settings, mental health professionals were more likely to be working in isolation and many were working remotely from home. Most of the professionals in our sample talked about feeling well supported by colleagues, managers and supervisors, but nevertheless, many were often the only mental health professional providing support in a particular setting and most had only irregular contact with their professional support systems.

‘*In terms of doing the work itself I feel well supported and with clinical supervision there is a space there to talk about the impact of the work…But yeah, I think it might well be important for us to think moving forward about some more support for the support staff, in that sense.’* (Simon, clinical psychologist, telephone helpline)

This isolation was exacerbated for many by remote working and trying to juggle the competing demands of work and family commitments at home, alongside reduced opportunities to engage with social support and previously enjoyed activities outside of work due to social restrictions.

‘*COVID19 has had such a huge impact on all of us, it is hard to differentiate the impact of being more isolated from your colleagues, not seeing your patients face-to-face, having to manage a level of distress from remote working, from seeing the backlog of cases filling up in your service and then go home and find all your usual sources of relaxation and stress relief are denied to you and that everything feels so restricted and oppressive I think everyone has been in that sort of mindset myself included.’* (Lily, consultant psychiatrist, hospital)

### Self-sacrifice and subjugation of own needs

Nearly all the mental health professionals in our sample had made significant sacrifices to their own well-being in the course of doing this work. Participants described working many extra hours, in the evenings and at weekends, and being less available for friends and family. This seemed to mirror the HSCWs they were supporting who were working above and beyond their normal requirements, and a sense that the mental health professionals therefore should too.

‘*We don't always apply it [to ourselves] I think it's been heightened by the fact that it's a crisis and there was a real kind of energy around doing, you know doing something and helping, and I think seeing our colleagues going above and beyond, I think that makes it harder to go actually I am going to be really precious, I'm a psychologist so I'm not going to do this.’* (Katrina, clinical psychologist, care homes)

Participants talked about a lack of time for reflection in what was initially quite a reactive response to provide support. They also acknowledged feelings of guilt about prioritising their own needs.

‘*I think there is also a bit of guilt around it because you are thinking I am not a nurse or a frontline worker, I don't need time off.’* (Nicole, clinical psychologist, hospital)

Some participants talked about taking steps to attend to their self-care, in recognition of increased workloads and additional demands. However, it was striking that when asked what support they had put in place for their own mental well-being, many participants laughed and said this was not something they had considered.

‘*It's never crossed my mind to see anyone about this. Not once have I thought to myself, I should speak to someone. I 100% would tell everyone else to do it. I did! I said to people you should get some support. Funny!’* (Sydney, mental health occupational therapist, Nightingale hospital)

Most participants said they felt they would be able to access mental health support for themselves if needed, however, only one participant in our sample had formally sought support for themselves in recognition of their own distress. They had been offered one assessment session and one brief follow-up session. Another participant highlighted a concern about confidentiality for mental health professionals if they did seek support.

‘*I am an employee of the hospital so if I wanted to go forward for some support I could I guess but it would probably be from within my colleagues and that might be a bit tricky.’* (Polly, clinical psychologist, ICU)

### Vicarious traumatisation and vicarious moral injury

Like their HSCW colleagues, mental health professionals also experienced traumatic and morally injurious events, both directly and indirectly through the health and social care colleagues they were supporting.

#### Traumatic exposure

Mental health professionals were exposed to hearing about many of the traumatic experiences that their health and social care colleagues had been through. For many mental health professionals, this was a context that they were not familiar with working in, and content that they were not used to being exposed to.

‘*I was on the ward one night when someone passed away. And I think for psychology, although we are used to suicides, that's only happened once in my career and I wasn't there to witness it. So, this was a very, very different way of working for me so yeah, it's been very emotional, I almost don't know where to start in terms of making sense of everything.’* (Katie, clinical psychologist, hospital)

What they were hearing in their support sessions also mapped on to mental health professionals’ own anxieties and triggers.

‘*We're also operating in the same shared traumatic reality as everybody else so you can find yourself really thinking about that person's experience of when they developed COVID and you can also have the same anxiety about developing COVID…I would say sometimes there's stuff in there that could be vicariously traumatising really.’* (Olly, trainee clinical psychologist, hospital)

Working in isolation meant that mental health professionals were often left to contain difficult emotions and content from their sessions with the HSCWs.

‘*There were times of feeling like you you're containing a lot of emotions from other staff on the helpline and then you kind of I guess you feel that you're left with quite a lot.’* (Laura, clinical psychologist, online staff support and telephone helpline)

Working remotely at home also meant that it was harder for many mental health professionals to maintain boundaries between work and home life.

‘*Something I found hard was the room I work in is also my bedroom. It can be a lot to have these difficult conversations in your own room where your bed is, not having that space.’* (Estelle, PWP, IAPT)

#### Ethical and moral dilemmas

Like their HSCW colleagues, mental health professionals often struggled with not always being able to do something to help.

‘*There are some things that you can't do…So for some you can feel that, yeah, I can be helpful to someone, but other times it feels that there is not much I can do for you other than hear you out.’* (Jade, mental health practitioner, ambulance service)

Mental health professionals also had to make difficult decisions about who would get their services and who would not.

‘*We also equally had the same experiences with moral injury, having to decide who to provide psychological support to and who not to because some people were using the service, or being referred to the service, despite not having issues related to the frontline. We've had to say we can't offer them support; we've had to prioritise people which also led us having to make quite difficult decisions.’* (Archie, trainee clinical psychologist, IAPT)

Finally, also like their HSCW colleagues, they often struggled with guilt about previous vulnerable patients who had been rapidly discharged or placed on waiting lists, while they were required to prioritise staff support.

## Discussion

In this study we set out to explore UK mental health professionals’ experiences, views and needs while working to support the well-being of front-line HSCWs during the COVID-19 pandemic. We found that mental health professionals were motivated to support HSCWs who they perceived as colleagues and doing so gave them meaning and purpose. Many experienced professional growth. However, this also came at significant costs.

The rapid requirement to step into new areas and set up new services in the absence of previous experience of, and an established evidence base for, treating front-line workers created anxiety and uncertainty and led to wide variation in service provision.

Mental health workers also struggled with blurred boundaries as their colleagues became their clients. Like the HSCWs they were supporting, mental health workers took on additional responsibilities and increased workloads and tended to neglect their own health and well-being in the line of their work. Unlike their HSCW colleagues, mental health professionals were frequently working independently and often remotely from home, resulting in professional and personal isolation. Mental health professionals were affected vicariously by the traumas and moral injuries that healthcare workers talked about in sessions, as well as directly by difficult decisions they had to make.

This crisis has also highlighted a paucity in high-quality research on supporting front-line HSCWs, as well as a dearth of recent research into the mental health of mental health professionals. This raises two key implications: the need for improved mental health support for HSCWs, and the need to attend to the mental health of the mental health professionals providing such support.

### The need for improved mental health support for HSCWs

The COVID-19 pandemic has underlined the need to provide more psychological support for front-line HSCWs. In October 2020, NHS England and NHS Improvement announced an investment of £15 million to fund rapid mental health assessment and treatment for NHS staff.^9^ This will include piloting of a series of specialist mental health hubs as well as access to more specialist treatment. This builds on the commitment outlined in the NHS People Plan 2020–21 published in July 2020, to provide a more supportive working environment for staff in the NHS.^10^

The findings of this study have identified that service provision during the pandemic varied widely, with most mental health professionals lacking specific knowledge of health and social care settings and the unique needs of staff. The investment in future support services for HSCWs will be benefitted by better communication, coordination and cohesion between mental health services. The development of such services will need to be informed by the growing evidence base about the effectiveness of staff support interventions and mental health professionals will be instrumental in developing this. The COVID-19 pandemic has accelerated our understanding of these issues and provides an opportunity to design support services that best meet the needs of the HSC workforce, and that are acceptable, accessible and equitable.

The provision of such services will rely on a mental health workforce that is adequately trained, resourced and well enough themselves to provide this support. Investing in mental health services for HSCWs therefore also requires investment in the mental health professionals who will be providing them. Such investment will require training of mental health professionals in working with high-risk occupational groups such as HSCWs; particularly considering their unique working context and needs, and managing issues such as confidentiality, empathy and vicarious traumatisation. Such training should be embedded in core professional training but also considered in regular professional development.

Such mental health services will also need to be sufficiently resourced to ensure that the mental health professionals within them are able to provide a high-quality and easily accessible service for HSCWs without risking burnout themselves.

### The need to attend to the mental health of mental health professionals

The findings of this study suggest that mental health professionals themselves may be at risk of adverse mental health outcomes. There is a lack of coherent evidence about the prevalence of mental health difficulties in mental health professionals. What research there is has suggested that treating clients affected by trauma can lead to high rates of compassion satisfaction, but also compassion fatigue, secondary traumatic stress and vicarious traumatisation.^11–14^ Compassion fatigue, secondary traumatic stress and vicarious traumatisation have all been recognised as pathways to occupational burnout with between 21% and 67% of mental health professionals reported to experience burnout at work.^15,16^

The mental health professionals in our sample felt a very strong sense of empathy with a workforce they defined as their colleagues. This fuelled their motivation to undertake this work and gave them a sense of purpose and meaning. However, empathy is a well-established vehicle for vicarious traumatisation^17^ and highlights how mental health professionals could be at particularly high risk for vicarious traumatisation in the current context. Research has also shown that health professionals are especially vulnerable if experiencing the same traumatic exposure as their clients, as evidenced by studies on 9/11^18^ and Hurricane Katrina.^19,20^

The current study has also shown that factors that may usually mitigate the risk of adverse mental health outcomes among mental health professionals have been compromised in the current pandemic context; including reduced contact with supervisors and peers, personal and professional isolation, lack of opportunity to engage in stress-reduction activities because of social restrictions, and lack of attention to and prioritisation of self-care. This in combination highlights an urgent need to attend to the mental health and well-being of the mental health professionals who are supporting the front-line workforce.

All of the participants in this sample reported having access to some form of supervision, but many did not access it, and most did not bring up their own well-being and support needs. Given the extent of self-sacrifice seen in this sample, alongside the notable lack of prioritisation of their own mental health, we should be reminded of the importance of supervisors regularly checking in with their supervisees and ensuring that discussing their well-being is a priority. This may be particularly crucial when mental health professionals are impacted personally, as well as professionally, by their work. Supervision of supervision will be imperative in ensuring that this is supported.

### Key recommendations

Our key recommendations are as follows:

- Well-being support for mental health professionals should be prioritised, frequently monitored and integrated into regular support structures, including supervision and supervision of supervision.
- Training in the work context and the needs of HSCWs, as a high-risk occupational group, should be included in mental health professionals’ training.
- Developing an evidence base for effectively supporting HSCWs is urgently needed and should be co-produced with HSCWs but also with the mental health professionals who will be called on to deliver this.
- More research is needed on the mental health of mental health professionals.

### Strengths and limitations

We deliberately sought to include a diverse group of mental health professionals from all regions of the UK in the sample, including participants from different professional groups and different career stages. This has enabled us to explore the range of mental health professionals’ experiences and thereby to increase the potential transferability of our findings. Our research team was diverse and included clinical academics with considerable NHS experience. The perspectives of mental health professionals who were working in settings supporting HSCWs were included in the design, delivery, analysis and write up of this paper. The qualitative methodology employed in this study was rigorous, with all steps taken throughout to maximise the validity and trustworthiness of the findings.

This study nevertheless has certain limitations. We did not record the age or ethnic origin of participants at the point of recruitment. Our sample consisted of participants from a range of career stages, so likely representing a variety of ages. However, we cannot comment on the ethnic composition of our sample nor therefore the potential transferability of our findings to mental health professionals of different ethnic backgrounds. Although we achieved a good range of participants from throughout the UK, our final sample was quite London-centric, partly due to one method of recruitment being snowball sampling through health and social care contacts of a London-based research team. Although we noted themes that were relevant across geographical areas of the UK, the nuances of people's experiences in different UK regions does warrant further research.

Our sample also contained a large proportion of clinical psychologists (46%). Although this is not necessarily unrepresentative of the population of mental health professionals tasked with providing support to workers in hospital and social care settings, further research exploring the experiences and views of other mental health professional groups during the pandemic would be helpful.

### Implications

This study provides an in-depth insight into the experiences, views and needs of mental health professionals who have been tasked with providing support for front-line HSCWs during the COVID-19 pandemic in the UK. Studies into COVID-19 and plans for its management have so far largely overlooked the impact on the mental health workforce. We found that mental health professionals were very motivated to step up and support their HSCW colleagues, and many derived meaning and growth from this work. However, this also came at some costs as they took on additional responsibilities and increased workloads, were anxious and uncertain about how best to support this workforce and tended to neglect their own health and well-being. Many were professionally isolated and affected vicariously by the traumas and moral injuries that healthcare workers talked about. This research highlights the urgent need to pay attention to the mental well-being, training and support of the mental health workforce who are supporting front-line workers.

## Data Availability

The data that support the findings of this study are available from the corresponding author (J.B.), upon reasonable request. The data have not been made publicly available due to the personal and sensitive content of the participants’ accounts.

## Supplementary material

For supplementary material accompanying this paper visit http://dx.doi.org/10.1192/bjo.2021.29. click here to view supplementary material
