# Supplementary material for: Experiences of mental health professionals supporting front-line health and social care workers during COVID-19: qualitative study
Source: BJPsych Open. 2021 Mar 23;7(2):e70. doi: 10.1192/bjo.2021.29 (PMC8007934; doi:10.1192/bjo.2021.29)
Supplement: Supplementary file 1 [file S2056472421000296sup001.docx]

**Supplementary Material**

**Supplement Appendix 1**

**Interview Schedule – Mental Health Workers**

Thank you for agreeing to take part in this interview. The interview should last for about 30 minutes. You can take a break at any time or if you would like to postpone the interview, please do just let me know. If at any point you want to stop the interview and no longer take part, then that is fine too, please just say. Please try to avoid mentioning any identifying features of your colleagues or place of work. If you do, don’t worry, we will just make sure to remove this from the interview transcript. This interview is completely confidential and anonymous, no information about you, your colleagues or your place of work will be identifiable. Are you happy to continue? Thank you.

1. Can you tell me a bit about your usual role?
   - *Where do you usually work?*
   - *What do you usually do in your day to day work?*
2. Tell me about your current role and what work you are doing to support frontline healthcare professionals at the moment?
   - *How did this role come about? (i.e. were you redeployed, did you volunteer to do this?)*
   - *If so, what was the process of redeployment like for you?*
   - *What kind of psychological support are you providing?*
   - *In what format are you providing this? (in person vs remote? Group vs individual?)*
   - *What kind of problems are people bringing up?*
3. What has been your experience of providing support for frontline healthcare professionals so far?
4. Can you tell me about how you have felt when doing this work?
5. What are some of the challenges in doing this work?
6. Do you have all the skills and resources you feel you need to do this work?
   - *Is there anything more which could have been done to prepare you for doing this work?*
7. Do you feel like you have enough support to do this work? (e.g. supervision, consultation)
   - *If you feel supported enough, what is contributing to this?*
   - *If you feel you need more support, what would that look like?*
8. What sort of support do you think is most helpful to provide from your experience so far?
   - *Are there things that should be provided but aren’t?*
   - *Are there things being provided which you think shouldn’t be?*
9. What has been the impact on you of doing this work?
10. What has helped you to cope with doing this work?
11. Have you been offered any psychological support yourself?
12. Has the pandemic affected you in your personal life as well?

- *If so, how has this interacted with you doing your work?*

1. What are your thoughts about how the media has represented healthcare workers during the pandemic?
2. Has there been anything unexpected/that has surprised you in doing this work?
   - *Anything positive? Unexpected gains?*
3. What support do you think you (and your team) might need going forward?
4. If you were put in charge of the provision of psychological support for healthcare staff what would you recommend?
   - *What types of support?*
   - *Timing of support?*
   - *Delivery of support – i.e. by whom, where?*
5. Is there anything else you would like to tell us?

Thank you for your time. Do you have any questions?
